# Macrophage activity at the site of tumor ablation can promote murine urothelial cancer *via* transforming growth factor-β1

**DOI:** 10.3389/fimmu.2023.1070196

**Published:** 2023-01-24

**Authors:** Yasushi Kimura, Masashi Fujimori, Neeraj Raghuraman Rajagopalan, Krish Poudel, Kwanghee Kim, Karan Nagar, Laurien GPH. Vroomen, Henning Reis, Hikmat Al-Ahmadie, Jonathan A. Coleman, Govindarajan Srimathveeravalli

**Affiliations:** ^1^ Department of Diagnosis and Interventional Radiology, Osaka University Graduate School of Medicine, Suita, Osaka, Japan; ^2^ Dept. of Mechanical and Industrial Engineering, University of Massachusetts Amherst, Amherst, MA, United States; ^3^ Department of Radiology, Mie University, Tsu, Japan; ^4^ Division of Urology, Department of Surgery, Memorial Sloan Kettering Cancer Center, New York, NY, United States; ^5^ Department of Radiology and Nuclear Medicine, Amsterdam University Medical Center (UMC), Amsterdam, Netherlands; ^6^ Department of Pathology, Memorial Sloan Kettering Cancer Center, New York, NY, United States; ^7^ Institute for Applied Life Sciences, University of Massachusetts, Amherst, MA, United States

**Keywords:** ablation, macrophages, transforming growing factor-β1, irreversible electroporation, bladder cancer

## Abstract

Cell death and injury at the site of tumor ablation attracts macrophages. We sought to understand the status and activity of these cells while focusing on transforming growth factor-β1 (TGF-β1), a potent immunosuppressive and tumorigenic cytokine. Patients with urothelial cancer who underwent ablation using electrocautery or laser demonstrated increased infiltration and numbers of CD8+ T cells, along with FoxP3+ regulatory T cells, CD68+ macrophages and elevated levels of TGF-β1 in recurrent tumors. Similar findings were reproduced in a mouse model of urothelial cancer (MB49) by partial tumor ablation with irreversible electroporation (IRE). Stimulation of bone marrow derived macrophages with MB49 cell debris produced using IRE elicited strong M2 polarization, with exuberant secretion of TGF-β1. The motility, phenotypic markers and cytokine secretion by macrophages could be muted by treatment with Pirfenidone (PFD), a clinically approved drug targeting TGF-β1 signaling. MB49 cancer cells exposed to TGF-β1 exhibited increased migration, invasiveness and upregulation of epithelial-mesenchymal transition markers α-Smooth Muscle Actin and Vimentin. Such changes in MB49 cells were reduced by treatment with PFD even during stimulation with TGF-β1. IRE alone yielded better local tumor control when compared with control or PFD alone, while also reducing the overall number of lung metastases. Adjuvant PFD treatment did not provide additional benefit under *in vivo* conditions.

## Introduction

Urothelial cancer in the bladder and upper urinary tract is diagnosed at an early and localized stage in more than 70% of patients with the disease for whom preference is given to urinary function sparing focal therapies. Urothelial tumors are treated by ablation using laser, electrocautery fulguration or photodynamic therapy (1–4). Local tumor recurrence is commonly observed in these patients (1, 2, 5). The etiology of recurrence is not well understood where the relative contribution of incomplete tumor ablation and disease characteristics has not been delineated. Emerging ablation modalities such as irreversible electroporation (IRE) and vascular-targeted photodynamic therapy (VTP) have been studied to improve control of urothelial cancers (6–10). The working principle of these non-thermal ablation techniques does not require sustained alteration of tissue temperatures for tumor destruction, allowing penetrative application in the genitourinary tract with minimal risk of adverse events. While such techniques can improve the efficacy of focal ablation, there may exist other biological factors that contribute to local recurrence of urothelial cancers.

Tumor ablation with thermal and non-thermal techniques elicits a localized inflammatory response and immune cell activation (11). Tumor cells injured or killed by ablation release damage associated molecular patterns (DAMPs) and tumor antigens, promoting immune cell infiltration that can also augment anti-tumor response at distal sites (12–15). Immune-stimulation by ablation alone is generally insufficient for elimination of local-residual, or distant tumor burden, where adjuvants are required to facilitate anti-cancer immunity (16). Preclinical studies have shown increased efficacy when tumor ablation is combined with immune checkpoint inhibitors (ICIs) (17–21). However, preliminary clinical trials testing combination therapy with ablation and ICI have yielded modest results (22, 23). The mechanisms that hinder the efficacy of this combinatorial therapy approach is yet to be established.

In addition to immune cell stimulation, the site of ablation undergoes prolonged wound healing, scar formation and regeneration (15, 24, 25). Macrophages and fibroblasts, along with several other type of cells release cytokines during the wound healing and remodeling process. This includes transforming growth factor-β1 (TGF-β1) (24, 26, 27) that mediates fibroblast activation, collagen production and tissue homeostasis (28). In addition to its role in wound healing, TGF-β1 is also a potent immunosuppressive and tumorigenic cytokine that is exuberantly secreted by macrophages having M2 phenotype (29, 30). TGF-β1 exhibits differential interaction with cancer cells, acting as a tumor suppressor in the early stages of tumorigenesis but enhancing tumor cell survival and invasive behavior in the later stages of cancer development (31, 32). It is unclear whether urothelial tumor ablation with IRE would evoke similar outcomes.

The objective of this study was to understand macrophage activity in urothelial tumors following ablation with IRE, and the effect of TGF-β1 secretion by these cells on residual cancer cells in the tumor microenvironment. We also evaluated the benefit of modulating TGF-β1 signaling with a small molecule drug that has received approval for patient use (Pirfenidone, PFD).

## Materials and methods

### Patient information and samples

Biopsy specimens at initial diagnosis and recurrence were selected from non-consecutive patients with urothelial cancer in the bladder or distal ureter (n = 5, 1 male and 4 female, median age at intervention: 73.2 years; range: 66 – 91 years) treated with ablative therapies (laser or fulguration with electrocautery). Immuno-profiling of the tumor microenvironment was performed by immunohistochemistry for T cell makers (CD8, Dako, clone C8/144B), macrophages (CD68, Agilent/Dako, clone KP1), regulatory T cells (FoxP3, Abcam, clone 236A/E7) and transforming growth-factor β1 (TGF-β1). IHC stained slides were scanned at 20x magnification and used to quantify total number of cells or protein staining in the samples using an image processing software, QuPath (33).

### Cell line and cell culture

Murine urothelial cancer (MB49) cell line was maintained in Dulbecco modified Eagle medium (DMEM, Gibco Laboratories, Grand Island, NY) supplemented with 10% fetal bovine serum (FBS) (Gibco) and antibiotics (Antibiotic-Antimycotic, Gibco) at 37°C with 5% CO2. MB49 cells between passage 8-12 were used. MB49 cells were harvested by detaching with TrypLE™Express Enzyme (Gibco) from 10cm dish or T75 flask.

### Bone marrow derived macrophages generation

Bone marrow was harvested from 8–12 week-old healthy C57BL/6 mice (Charles River, Wilmington, MA) following established protocols to produce BMDMs (34). In brief, bone marrow was harvested from the femur and tibia, red blood cells were lysed using RBC Lysing Buffer (BioLegend, San Diego, CA) and the remaining cells were cultured in RPMI 1640 (Gibco) supplemented with 10% FBS, 1% antibiotics and 20 ng/mL macrophage-colony stimulating factor (mouse recombinant M-CSF, BioLegend) in 5% CO_2_ at 37°C. On day 3, the culture medium was replaced with fresh medium containing M-CSF. BMDMs were harvested after 7 days of M-CSF induced macrophage differentiation.

### Reagents

Pirfenidone (PFD) was purchased from Selleck Chemicals (Houston, TX) and was dissolved in dimethyl sulfoxide (DMSO) (Millipore Sigma, St. Louis, MO) for a final a concentration of 40mg/ml. The PFD solution was diluted with DMEM to a final concentration of 200µg/ml for *in vitro* experiments. TGF-β1 (Recombinant Human TGF-β1) was purchased from PeproTech, Inc (Rocky Hill, NJ), and dissolved in DMEM to 5ng/ml for in-vitro experiments.

### BMDMs stimulation

Harvested BMDMs were plated in 96-well plates (5x10^4^cells/well), 24-well plates (2.5x10^5^ cells/well) or 12 well-plates (5x10^5^ cells/well) and incubated at 37˚C with 5% CO_2_. Following attachment to the plate, the cells were serum starved overnight prior to stimulation studies. BMDMs were stimulated by exposure to MB49 cell debris. MB49 debris to BMDMs ratio was 1:1. MB49 cell debris was produced by treating suspension cells with IRE by exposure to electric pulses (1500V/cm, 90 pulses, pulse length of 100μs, 1Hz, repeated twice) in a 4mm gap cuvette. The MB49 cell debris was centrifuged at 2000rpm for 5 min following treatment with electric pulses. The debris pellet was re-suspended in DMEM and added to the wells. Equivalent volume of DMEM was added to wells designated for sham treatment. PFD (1mM) was added to wells designated for treatment at the same time. An equivalent volume of DMSO (1.5μL) was added to cells designated for sham PFD treatment.

### Cell viability assay

Cell Counting Kit-8 (CCK-8) assay (Dojindo Laboratories, Kumamoto, Japan) was used to measure cell viability. CCK-8 solution (10μL/well) was used to measure viability of samples in 96 well plates, followed by incubation for 4 hours at 37°C. The absorbance at 450 nm was determined by a multiplate reader (SpectraMax, Molecular Device). Cell proliferation was expressed as a percentage of the control group (untreated cells).

### Transwell migration assay

BMDMs (5x10^4^ cells/insert) were suspended in 100µl of DMEM with 10% FBS and plated in the upper chamber of a Transwell insert (24well, 8µm pore size; Corning Life Science, MA, USA). BMDMs were serum starved overnight prior to the migration assay. DMEM with 1% FBS was used as the chemoattractant. BMDMs stimulation by debris was performed in the inserts as described above. Following 24 hours of incubation at 37˚C with 5% CO2, the migrated cells were stained with Crystal violet (Millipore Sigma), and the number of cells on the lower side of the transwell insert were counted in 5 locations per sample with inverted microscope (100x, Axio Vert.A1, Carl Zeiss, German).

### Flow cytometry

Flow cytometry was performed to identify polarization of BMDMs following stimulation with cancer cell debris, with or without PFD treatment. BMDMs were seeded in a 12 well and stimulated as described above. To produce positive controls, the BMDMs were incubated for 24 hours with 20ng/ml mouse recombinant Interferon-gamma (IFN-γ) and 100ng/ml LPS for M1, or 20ng/ml mouse recombinant IL-4 for M2 phenotype respectively. Following stimulation, BMDMs were washed with PBS, centrifuged and triple stained for Pacific blue-CD11b (BioLegend, Dedham, MA, USA, Cat #101224), FITC-CD80 (BioLegend, Cat # 104706) and APC-CD206 (BioLegend, Cat #141708). BMDMs polarization was then assessed using a ACEA Novocyte flow cytometer and analyzed using NovoExpress 1.2.5 software.

### TGF-β1 measurements

BMDMs were seeded in a 12 well plate followed by stimulation with MB49 cell debris as described above. Following 24h of stimulation, cell culture supernatant was collected to measure TGF-β1 level by enzyme-linked immunosorbent assay (ELISA) using a Quantikine kit (R&D Systems, Minneapolis, MN) according to manufacturer’s instructions.

### Wound scratch assay

MB49 cells were plated and grown to 90% confluence in a 6-well plate. The medium was exchanged with PBS, and a straight scratch was made using a pipette tip (standard 1000µL pipet tips, Corning^®^ DeckWorks™, Corning Life Science, MA, USA). Wells were rinsed 3 times with PBS immediately after scratching, and DMEM containing 1% FBS with or without 5ng/ml of TGF-β1, and/or 200µg/ml of PFD was added to the samples. Cells were incubated at 37˚C with 5% CO_2_ and phase contrast microscope images were obtained at the scratch locations at 0, 6, 12, and 24 hours after wound creation. The gap between the monolayers was measured at 10 scratch locations in each well, and the half of mean distance of scratch closure was calculated as the migration distance.

### RT-PCR preparation

MB49 cells were plated in 24 well plates and serum starved overnight. TGF-β1 stimulation and PFD treatment was performed as described above. Each experiment included three biological replicates per treatment condition. Following 24h incubation, each well was washed with PBS twice and RNA was extracted using Tryzol (Invitrogen, Carlsbad, CA, United States). Extracted RNA was directly purified with the Zymo Research Direct-Zol RNA MicroPrep kit. Approximately 500 ng of RNA was used to create cDNA with SuperScript IV Reverse Transcriptase, RNaseOut, 10 mM dNTPs, and 50μM Random Hexamers (ThermoFisher, Pittsburgh, PA), with a sample volume of 20μL. The cDNA was frozen at −20°C and then used for RT-PCR within 1 week. RNA and cDNA quantities were measured using a NanoDrop 2000 (ThermoFisher).

### Quantitative RT-PCR

RT-PCR was performed with cDNA as prepared above using a CFX Connect real-time system (Bio-Rad) with iTaq Universal SYBR Green Supermix (Bio-Rad, Hercules, CA). Primers for β-actin, vimentin and α-SMA were purchased from Bio-Rad. Relative gene expression was determined by comparing the Ct value of the gene of interest to that of the β-actin as housekeeping gene, by the 2ΔΔCt method. Three technical replicates were performed for each biological sample. There was no amplification for the no-template control (NTC). Data was analyzed using the CFX Maestro Software. Ct values were generated using the point at which the sample fluorescence value exceeded the software’s default threshold value. Each sample was normalized to the untreated control.

### Mouse experiments

All animal experiments were performed following protocols approved by the Institutional Animal Care and Use Committee. MB49 cells were injected subcutaneously into right flank of 10–12 week-old male C57BL/6 mice (Taconic). The mice were randomized to the following four groups once the tumors reached 5mm in any one dimension: Control, IRE, PFD, and IRE + PFD (each, n=16). IRE using a caliper electrode (Tweezertrodes) and a square wave generator (ECM830 Electroporation System, Holliston, MA). IRE for complete tumor ablation was performed with 2000 V/cm, 90 pulses, pulse length of 100μs, 1Hz. For sub-total (partial) ablation, IRE was performed with 1250 V/cm, 50 pulses, pulse length of 100μs, 1Hz was administrated. PFD was administered by intraperitoneal injection (200 mg/kg) starting the day after IRE treatment and continued for two weeks. Animals treated with IRE for partial ablation were sacrificed on 2 and 9 days (n = 4 each) following treatment, with matched mice from the control group. Tumors from these animals were collected for histological analysis. Animals receiving complete IRE ablation were monitored for treatment response. Tumor volume (TV) was calculated as the product of a maximum tumor diameter (D) and short diameters (d’, d’’) which are perpendicular to each other: TV = D x d’ x d’’. Mice were euthanized per institutional guidelines when the tumor volume exceeded 1.5 cm^3^, or if the animals demonstrated morbidity. The flank tumor and both lungs were collected for histological analysis. Three cross sections of lung specimens from each mouse were H&E stained, and the number of metastases in each sample, and area of the tumor as percentage of lung in the slide was quantified with QuPath.

### Immunohistochemistry

Tumor and lung tissue were formalin fixed and paraffin embedded. Sections were stained for CD8 (CST98941, rabbit anti-mouse), F4/80 (macrophage marker, eBioScience BM8, rat anti-mouse), FoxP3 (CST12633, rabbit anti-mouse) and TGF-β1 (ab215715, rabbit anti-mouse). Stained slides were scanned at 4x and 20x magnification, and cells or tissue positive for markers in field of view was quantified with QuPath.

### Statistical analysis

All *in vitro* experiments were duplicated, and data were showed as mean ± standard deviation (SD) unless otherwise indicated. The student t-test were used to compare the results of CCK-8, 3D migration assay, flow cytometry, ELISA, scratch assay, RT-PCR, tumor response and histological analysis. Overall survival rates were calculated based on the Kaplan-Meier method and compared with log-rank test. All statistical analyses were performed using GraphPad Prism 8 (GraphPad Software Inc., San Diego, CA). A p-value of <0.05 was considered as statistically significant.

## Results

### Patients with recurrent urothelial tumors have greater levels of CD8+ cells in an immunosuppressive environment

**Figure 1:**
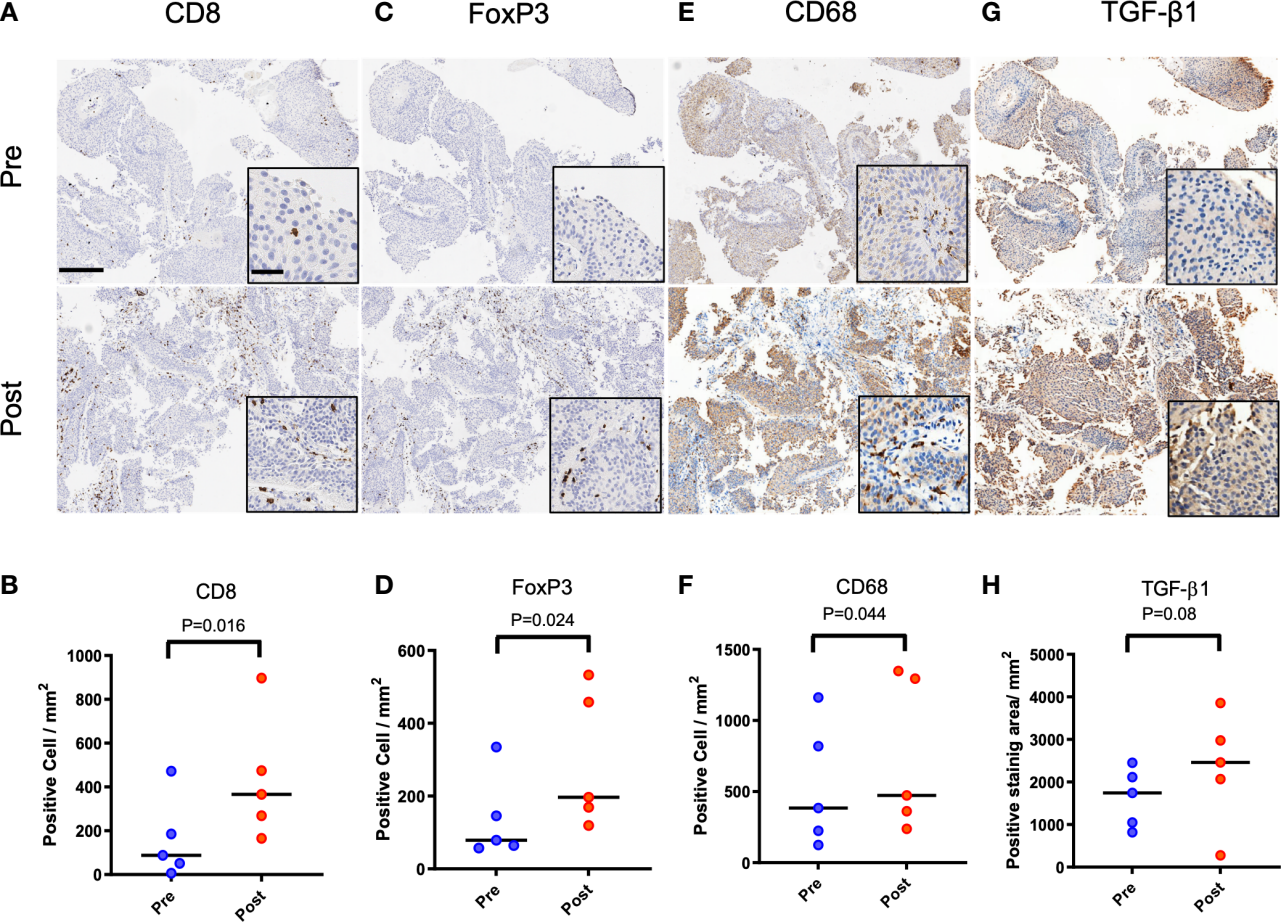
Immune infiltrates and TGF-β1 expression in primary and recurrent urothelial cancer in patients initially treated with ablation. Patient 1 (female, 69 years) with biopsy confirmed urothelial tumor treated with resection, laser ablation and electrocautery. Tumor recurrence was biopsy confirmed at 3 months following initial treatment. **(A, B)** CD8 positive cells at recurrence was higher than at initial diagnosis (P=0.016). **(C, D)** FoxP3 positive cells in samples increased at recurrence (P=0.024). **(E, F)** More CD68 positive cells was confirmed after recurrence (P=0.044). **(G, H)** TGF-β1 expression positive area was also increased without statistical difference (P=0.08). Scale bar indicates 250μm in low magnification and 50μm in high magnification images (inset).

Patients (n = 5) diagnosed with urothelial cancer and having recurrence after initial treatment with ablation were selected at random for evaluation of their biopsy samples with immunohistochemistry. Two patients had tumors in their ureter while the tumors for the other three patients were located in the bladder. Four patients were treated by transurethral tumor resection and electrocautery, while one patient with a ureteral tumor underwent laser ablation. Time to tumor recurrence ranged from 2-12 months. Recurrence was not at the site of the treated preliminary tumor. Compared to biopsy at initial diagnosis, samples taken at recurrence had 2-fold greater numbers of CD8+ T cells (**Figures 1A, B**, P<0.05) by immunohistochemistry. Similar increase in the numbers of FoxP3+ regulatory T cells was noted (**Figures 1C, D**, P<0.05). Comparison of biopsy samples from the same patient indicated a 37% increase in the presence of CD68+ macrophages (**Figures 1E, F**, P<0.05) and 42% increase in the biopsy area staining positive for TGF-β1 (**Figures 1G, H**, P=0.08).

### Partial ablation of urothelial tumors with IRE reproduces immuno-microenvironment found in patients with post-treatment urothelial cancer recurrence

**Figure 2:**
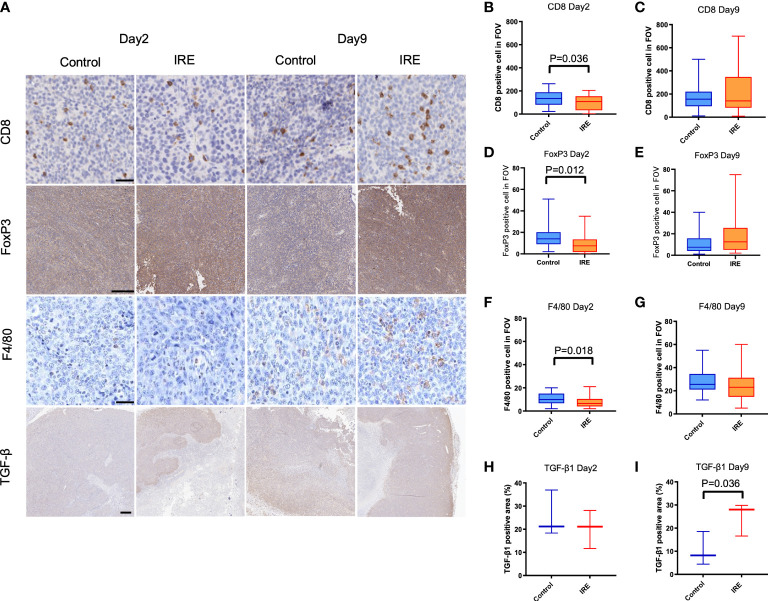
Immune infiltrates and TGF-β1 expression in sham control and partial IRE of subcutaneous MB49 urothelial tumors in mice. Histological samples were collected on Day 2 and Day 9 after IRE. **(A)** Representative histologic images showing: **(B, C)** CD8 positive cells on Day 2 were decreased after IRE compared to control (P=0.036, B), but recovered by Day 9. **(D, E)** FoxP3 positive cells on Day 2 were significantly decreased after IRE compared to control (P=0.012, D), but recovered on Day 9. **(F, G)** Partial IRE reduced F4/80 positive cells on Day 2 (P=0.018, F), then recovered on Day 9. **(H, I)** Compared to control, TGF-β1 expression positive area reduced on Day 2 without significance, but was significantly increased on Day 9 (P=0.036). Scale bar is 50μm in CD8 and F4/80, 250μm in FoxP3 and 500μm in TGF-β1.

Representative figures are shown in **Figure 2A**. Partial IRE induced approximately 50% or greater necrosis in tumors as seen on H&E staining. Despite this, residual tumor growth following partial IRE on day 2 (control: 51.4 ± 9.1 mm^3^; IRE: 45.9 ± 17 mm^3^) and day 9 post-treatment (control: 76.9 ± 17.1mm^3^; IRE: 60.3 ± 18.7mm^3^) were not significantly different (P=0.31). IRE treatment resulted in an immediate decrease of CD8+ T cells, F4/80+ macrophages and TGF-β1 on day 2 post-treatment, consistent with tumor ablation and necrosis. Similar to patient samples, partial IRE treatment stimulated the infiltration and numbers of CD8+ T cells by day 9 (day 2: 99.0 ± 66.3 cells/FOV vs. day9: 223.4 ± 188.1 cells/FOV, P<0.01, **Figures 2B, C**) to level that was 33% greater than sham control. Partial treatment with IRE also substantially increased numbers of FoxP3+ T cells on day 9 post-treatment (**Figures 2D, E**). IRE reduced intratumoral macrophages numbers (Sham: 11.0 ± 4.9 cells/FOV vs. IRE: 7.8 ± 5.1 cells/FOV, P<0.05, **Figure 2F**) on day 2 that then recovered to levels no different from sham controls on day 9 (Sham: 28.2 ± 10.2 cells/FOV vs. IRE: 24.9 ± 13.9 cells/FOV, P=0.30, **Figure 2G**). Partial treatment with IRE also decreased the TGF-β1 positive area on day 2 without significance (P=0.52, **Figure 2H**). Increased positive staining for TGF-β1 was observed on day9 when compared to control (IRE 24.8% ± 7.2 vs. 10.4 ± 7.3% of positive area, P<0.05, **Figure 2I**). FoxP3 cells demonstrated trends similar to macrophages (Control vs. IRE: 16.2 ± 10.8 vs. 9.4 ± 9.4, P<0.05 on day 2 and 12.1 ± 11.1 vs. 17.8 ± 17.0, P=0.12 on day 9).

### MB49 cell debris promotes M2 polarization and TGF-β1 secretion in BMDMs

**Figure 3:**
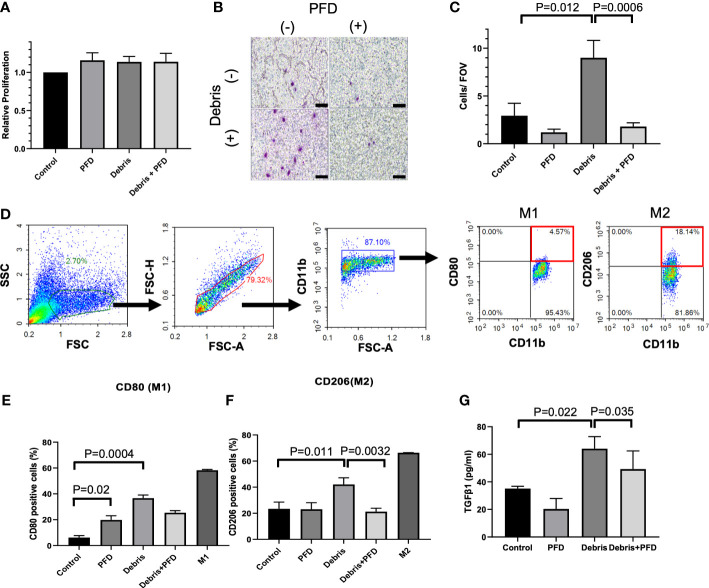
Outcomes of BMDM stimulation with IRE treated MB49 cells. **(A)** Stimulation of BMDM with tumor debris +/- PFD treatment did not alter relative cell viability. **(B, C)** BMDM stimulated with MB49 cell debris demonstrated greater migration in transwell assay (P=0.012) where treatment with PFD reduced this effect (P=0.0006). Scale bar is 100 μm. **(D)** Gating strategy used to identify BMDM subsets expressing M1 or M2 macrophage surface markers. After the exclusion of doublets and debris, macrophages were identified by CD11b staining, followed by the identification of sub-populations with expression patterns: M1 like macrophages (CD11b+, CD80+) and M2 like macrophages (CD11b+, CD206+). **(E)** Both debris and PFD were associated with higher CD80 positive macrophage sub-populations (P=0.02, and P=0.0004, respectively). **(F)** Stimulation with cell debris induced greater CD206 positive macrophage sub-populations (P=0.011). This shift in macrophage polarization was reduced by treatment with PFD (P=0.0032). **(G)** ELISA quantification of TGF-β1 secreted by BMDMs. Compared with control, BMDMs stimulated by debris secreted more TGF-β1 (P=0.022) and PFD suppressed this effect (P=0.035).

Compared to control, PFD, Debris and Debris + PFD did not impact macrophage proliferation as measured with the CCK-8 assay (**Figure 3A**). Stimulation with MB49 debris increased macrophage migration across the transwell insert with numbers (9.0 ± 1.8 cells/FOV) that were significantly higher than control (2.9 ± 1.3 cells/FOV, P<0.05) where migration was reduced by PFD treatment (1.8 ± 0.4 cells/FOV, P<0.001) (**Figures 3B, C**). The gating strategy is shown in **Figure 3D**. MB49 stimulation increased the population of both M1 (CD80+CD11b+) phenotype (Sham: 6.2 ± 2.5% vs. Debris: 36.7 ± 4.2%, p<0.0001, **Figure 3E**) and M2 (CD206+CD11b+) phenotype (Sham: 23.3 ± 5.2% vs. Debris: 42.1 ± 5.1%, p<0.01, **Figure 3F**) in comparison to sham control. PFD treatment in unstimulated macrophages did not impact M2 polarization levels, but it was associated with a slight increase in M1 polarization. PFD treatment substantially reduced both M1 and M2 polarized macrophages stimulated with MB49 debris. TGF-β1 secretion was increased by stimulation with MB49 debris (control; 35.1 ± 1.7 pg/mL, debris; 64.1 ± 8.8 pg/mL, P<0.05) while PFD reduced secretion of the cytokine in unstimulated and stimulated macrophages. (49.2 ± 13.2 pg/mL, P<0.05, **Figure 3G**).

### TGF-β1 promotes the invasiveness of MB49 cancer cells by epithelial to mesenchymal transition

**Figure 4:**
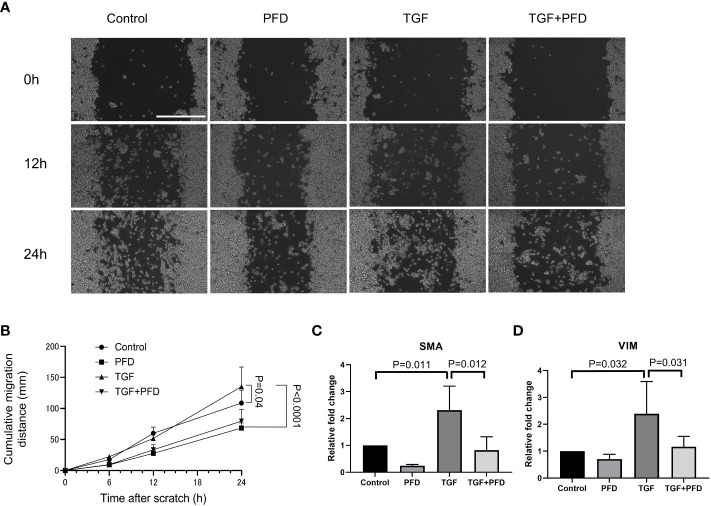
Effect of PFD treatment on MB49 migration and expression of epithelial to mesenchymal transition markers. **(A)** Migration distance on the cell culture plate was analyzed with scratch assay. Scale bar indicates 400μm. **(B)** MB49 cells incubated with TGF-β1 migrated significantly faster than control (108.8 ± 20.0µm vs. 135.3 ± 31.7µm, P=0.04). PFD treatment reversed the effect of TGF-β1 stimulation (79.6 ± 18.9µm, P<0.0001). **(C, D)** RT-qPCR quantification of EMT gene expression. Compared with control, both SMA and Vimentin expression were upregulated on exposure to TGF-β1 (P=0.011 and P=0.032, respectively), while PFD treatment suppressed this change in expression (P=0.012 and P=0.031, respectively).

Compared to control (**Figure 4A**), presence of TGF-β1 promoted quicker migration and wound gap closure in a scratch assay at 24h (Sham: 108.8 ± 20.8mm vs. TGF-β1: 135.25 ± 31.7 mm, P<0.05, **Figure 4B**). PFD alone impeded migration of cells (68.4 ± 8.4 mm, P<0.0001) and was effective in reducing cell motility even in the presence of exogenous TGF-β1 (79.6 ± 18.9 mm, P<0.0001). Compared with control, both SMA and Vimentin expression were increased in MB49 cells stimulated with TGF-β1 (relative expression; SMA: 2.3 ± 0.9, VIM: 2.4 ± 1.2, P<0.01 respectively, **Figures 4C, D**), which was muted by treatment with PFD relative expression; SMA: 0.8 ± 0.5, VIM: 1.2 ± 0.4, P<0.01 respectively). Western blotting for Vimentin did not show an appreciable change in protein abundance following TGF-β1 stimulation, or treatment with PFD at 24 or 48 hours (**Supplemental Figure 1**).

### Complete subcutaneous tumor ablation with IRE but not adjuvant PFD improves survival and reduces metastatic disease in mouse model of urothelial cancer.

**Figure 5:**
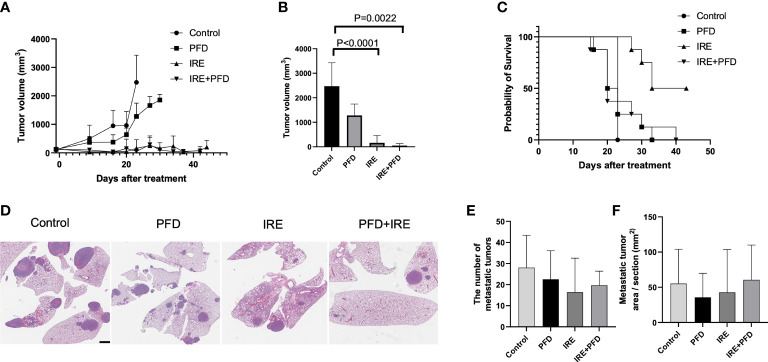
Tumor growth, survival and lung metastasis in mice bearing MB49 subcutaneous tumor treated with IRE +/- PFD. **(A)** Both IRE and IRE+PFD suppressed growth of the primary tumor when compared to control and PFD alone. **(B)** Mice in IRE and IRE + PFD group had significantly smaller tumor volume at the time of Day 23 (sacrifice date for all untreated mice) compared to control (P<0.0001 and P=0.0022, respectively). **(C)** Survival curve, mice in IRE group survived the longest. PFD monotherapy did not show survival benefit compared to control (p=0.5). PFD also showed no additional survival benefit alone or in combination with IRE (p=0.8). **(D)** Representative lung H&E-stained histology images showing metastasis. Scale bar is 2mm. **(E, F)** All treatments reduced distant metastasis in the lung without significant difference in the number of metastasis and metastatic tumor area pre section between groups.

As shown in **Figure 5A**, tumor growth was suppressed in IRE and IRE+PFD group when compared with control group and PFD group. Compared to control, mice in IRE group and IRE + PFD group had significant smaller tumor volume at the time of Day23 (Control; 2475 ± 954.9mm^3^, IRE; 163 ± 299.6mm^3^, P<0.0001, IRE + PFD; 51 ± 85.4mm^3^, P<0.01, **Figure 5B**). IRE + PFD group showed smaller tumor volume than IRE, but there was no significant difference (P=0.55). PFD monotherapy moderately inhibited the tumor growth compared to control (PFD; 1278 ± 468.3mm^3^, P*<*0.05). All the mice in control group were sacrificed on day 23 post-treatment to be consistent with protocol euthanasia guidelines. Compared to control, mice in IRE group significantly survived longer (P<0.001, **Figure 5C**). Besides, the addition of adjuvant PFD demonstrated no additional survival benefit (P=0.80). Representative images of lungs in each group are shown in **Figure 5D**. Quantification of lung metastases indicated that animals undergoing IRE showed fewer metastatic sites (16.4 ± 16.2 vs. 28.1 ± 15.3, P=0.06, **Figure 5E**) and reduced metastatic tumor area in lung (42.7 ± 60.8 mm^2^ vs. 55.3 ± 48.7 mm^2^, P=0.62, **Figure 5F**) compared to control. Compared to control, adjuvant PFD following IRE also provided moderate benefit in controlling the number lung metastasis (19.7 ± 6.7, P=0.16, **Figure 5E**) and reduced metastatic tumor area in lung (60.7 ± 49.2 mm^2^, P=0.43, **Figure 5F**). PFD alone had minimal effect on the number of metastatic sites (22.5 ± 13.5, P=0.27, **Figure 5E**), but the reduced metastatic tumor area in lung (35.8± 34.1 mm^2^ P=0.24, **Figure 5F**) when compared to control. There was no significant difference in the number lung metastasis and overall burden between treatment groups.

## Discussion

Macrophages are widely known to mobilize and infiltrate the site of ablation (15, 35, 36), secreting cytokines such as TGF-β1 that are classically associated with wound healing and regeneration (28, 37). However, TGF-β1 can also have pro-cancer effects in a context dependent fashion. Our *in vitro* studies demonstrate this phenomenon as BMDMs take on a wound healing phenotype (M2) when stimulated with cancer cell debris, secreting TGF-β1. This macrophage derived cytokine had a strong effect on urothelial cancer cells, mediating epithelial to mesenchymal transition with an associated increase in invasiveness. Under *in vitro* conditions, pirfenidone was able to mute both macrophage secretion of TGF-β1 and associated changes in cancer cells. However, adjuvant pirfenidone following tumor ablation with IRE did not improve local or distant metastasis control. Interestingly, complete elimination of the primary tumor with ablation had the greatest impact on metastases formation.

Ablation results in an internal wound that is remodeled and absorbed by the body over a prolonged period of time, lasting several months or even years in patients (15, 25, 38–40). Ablation related internal wounding skews macrophages to a M2 – or wound healing phenotype that shares several similarities with TAMs (41–43). Past literature indicates that such phenotypic polarization and activity of macrophages is largely independent of the ablation modality used or the tumor type being treated (15, 25). M2 macrophages secrete several cytokines that can have pro-tumorigenic effect (44–46), where pharmacologic modulation of these cytokines have shown some anti-cancer benefit in preclinical studies, including abscopal effects (47–51). In this work, we focused on TGF-β1 due to two reasons. First, clinical trials testing checkpoint immunotherapy in urothelial cancer has yielded promising preliminary results, where myeloid cells in the tumor microenvironment are associated with emergence of treatment resistance (52). Second, ablation is also known to stimulate immunity, yet ablation alone rarely elicits broad anti-cancer activity in patients. Analysis of patient derived initial and recurrent urothelial tumor samples connected these threads where we observed robust increase in CD8+ T cell infiltration in recurrent tumors following initial treatment with ablation, consistent with expectations of immune stimulation by ablation. Yet, there was concomitant increase in immunosuppressive cells and cytokines such as TGF-β1 in recurrent tumor microenvironment. In urothelial cancers, TGF-β1 signaling has been linked to tumorigenesis and increased epithelial-to-mesenchymal transition (EMT) (53, 54). In addition, TGF-β1 signaling negatively influences the tumor immune microenvironment by suppressing CD8+ T and natural killer (NK) cells, and by promoting regulatory T-cell (T-reg) proliferation (28, 55, 56). This immunosuppressive cytokine is also known to reduce the efficacy of immune checkpoint inhibitors in urothelial cancers (57–59). In addition to immunosuppressive effects, TGF-β1 also acts directly on established cancer cells by promoting their invasiveness and aggressiveness (60–63).

PFD is a small molecule drug approved for the treatment of patients with idiopathic pulmonary fibrosis. PFD has been shown to indirectly modulate the TGF-β1 pathway, with resultant anti-fibrotic effects (64). PFD is also known to exert direct anti-proliferative and suppressive effects on macrophages, though the mechanisms are not well understood (28). While most studies have evaluated the antifibrotic effect of PFD, the impact of PFD on macrophages and TGF-β1 is still being studied. As expected, our *in vitro* experiments demonstrated that PFD activity against macrophages by reducing their motility, M2 polarization and reducing TGF-β1 secretion. Likewise, exposure to PFD reduced the invasiveness of MB49 cancer cells, while also reducing expression of EMT related markers when exposed to TGF-β1. However, PFD proved less effective under *in vivo* conditions. Several reasons could have contributed to this effect. Even when carefully performed, ablation of subcutaneous tumors without image confirmation of immediate post-procedural treatment outcome can leave behind residual tumor burden. We anticipated that such tumor cells would be exposed to macrophages and TGF-β1, which would have downstream effects on metastasis formation. Our expectation was that adjuvant PFD would curtail these effects, with impact on reducing distant disease. It may be possible that PFD curtailed direct TGF-β1 signaling on tumor cells as seen *in vitro* but had minimal effect of immunosuppressive effect of the cytokine under *in vivo* conditions. Even if the immune effects of TGF-β1 were neutralized by PFD, ablation mediated immune response could have been muted by upregulation of checkpoints. We lack additional data and analysis to verify these hypotheses which would form the basis for future studies.

Interestingly, lowest metastatic burden was associated with effective local tumor control with IRE where adjuvant PFD did not demonstrate a strong benefit. Examining **Figures 5E, F** reveal that area of metastases and the total number in lung generally reduce with PFD treatment but significant outcomes were confounded by variations within groups. Our study was powered assuming that TGF-β1 would have sizeable impact on cancer cell invasiveness that mediated metastasis dissemination. However, it is possible that our assumptions underlying experimental design were not robust and that larger sample sizes are required to truly establish benefit. Moreover, it may be possible that total cancer burden present at the primary tumor plays a larger role in seeding metastasis than relative invasiveness of the cells. This would be consistent with the outcome that complete ablation of tumor with IRE reduced overall metastatic burden. While mechanisms underlying the blunted efficacy of PFD are unclear, the toxicity of combined therapy, limitations in PFD dosing regimen and bioavailability and other competing but unstudied factors may have contributed to this effect.

Our study provides intriguing preliminary evidence of macrophage activity in the post-ablation tumor setting, with links to TGF-β1. There are several limitations in a preliminary exploration such as ours. First, our study used single bladder cancer subcutaneous tumor model. Bladder immune microenvironment may be different from subcutaneous, where orthotopic tumors in the bladder may exhibit divergent tumor immuno-microenvironment. Our studies focused on a single cytokine, TGF-β1, whereas macrophages secrete several other cytokines (such as IL-10, M-CSF) which have immunosuppressive effects. Likewise, TGF-β1 can be secreted by other cells in the tumor microenvironment, such as endothelium, fibroblasts etc. Comprehensive profiling of the cell-cytokine interactions must be performed to fully understand post-ablation immunosuppression, but is not attempted here due to the inherent complexity of this task. Our choice of profiling tumors with immunohistochemistry limited capturing population level changes and cell-cell interactions that can be uncovered with flow cytometry. Further our preliminary exploration in patients was limited by numbers enrolled, the non-randomized status and demographic factors. These results are to be viewed as suggestive and not definitive for tumor immuno-environment following ablation in patients. Likewise, IRE is not standard of care for ablation of bladder tumors in patients. We used this technique as it was an interesting emerging tool that allowed ablation of tumors while preserving structures, thereby allowing examination of immune cell activity. Conventional ablation techniques such as electrocautery or laser evaporates the tumor bulk, with unpredictable levels of residual tumor for experimental purposes. We anticipate that IRE may have a role in treatment of urothelial cancer in future, especially as a tool to fully treat tumors while also priming the immune system, and in that context our findings are beneficial.

In conclusion, urothelial tumors ablation results in increased TGF-β1 levels in the residual tumor microenvironment from macrophage activity. Exposure of surviving cancer cells to TGF-β1 had pro-cancer effect, which was curtailed by PFD *in vitro*. Complete primary tumor ablation provided superior results when compared TGF-β1 modulation in the adjuvant or combinatorial setting.

## Data availability statement

The raw data supporting the conclusions of this article will be made available by the authors, without undue reservation.

## Ethics statement

The studies involving human participants were reviewed and approved by Waiver by IRB of Memorial Sloan Kettering Cancer Center. Written informed consent for participation was not required for this study in accordance with the national legislation and the institutional requirements. The animal study was reviewed and approved by IACUC of Memorial Sloan Kettering Cancer Center.

## Author contributions

*In vitro* experiments: MF, YK, NR. Image analysis and quantification: MF, YK, GS, KP, NR. Histology: HR, HA-A. Statistical analysis and results: MF, YK, GS. Animal experiments: MF, LV, KK, KN, and Manuscript preparation: MF, YK, JC, and GS. All authors contributed to the article and approved the submitted version.
