# Supplementary material for: Macrophage activity at the site of tumor ablation can promote murine urothelial cancer via transforming growth factor-β1
Source: Front Immunol. 2023 Jan 24;14:1070196. doi: 10.3389/fimmu.2023.1070196 (PMC9902765; doi:10.3389/fimmu.2023.1070196)
Supplement: Supplementary file 1 [file DataSheet_1.docx]

**Supplementary Information**

*Western bloting:* MB49 cells were treated with or without TGF-β1 (5 ng/mL) and/or PFD (200 µg/mL) in serum starved media (2% FBS) for 24 and 48 hours. Cells were lysed using M-PER buffer (Cat# 78503, ThermoFisher) with HALT protease inhibitor cocktail (Cat# 78443, ThermoFisher) and the protein concentration was measured using BCA assay (Cat # 23227, ThermoFisher). An equal amount of protein was used for each condition and mixed with laemmli buffer and boiled at 95 °C for 5 min. The resulting protein mixture is loaded into electrophoresis gels and run at a voltage of 200 V for 30 min. After electrophoresis, the protein is transferred from the gels to a PVDF membrane using a trans-blot turbo transfer system for 7 min. The blots are blocked using 5% non-fat dry milk for 1 h and washed thrice using tris buffered saline with 0.1% tween 20 (TBS-T) for 5 min each. The blots are incubated overnight at 4 °C with the primary antibody (anti β-Actin - 13E5 rabbit mAb – cat# 4970S; anti-vimentin (D21H3) rabbit mAb – cat# 5741S, Cell Signaling Technology) diluted in 5% BSA and then with secondary antibody conjugated to HRP (Anti-rabbit IgG, HRP-linked Antibody – cat# 7074S) diluted in 5% non-fat dry milk for 1h at room temperature. The blots are washed 5-6 times with TBS-T for 5 min each and then rinsed with TBS to remove the detergent completely. The HRP signal was developed using enhanced chemiluminescence (ECL) reagent and the blots were imaged using chemiDOC western blot imaging system.


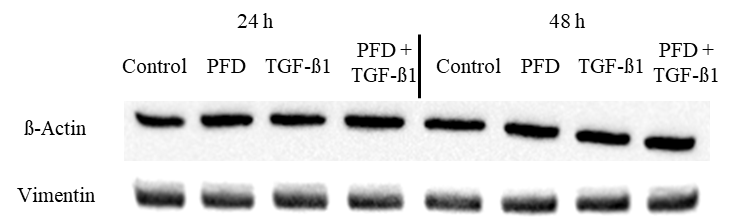


**Supplemental Figure 1.** Western blotting of Vimentin abundance in MB49 cells. Compared to unstimulated cells, TGF-β1 with or without PFD treatment elicited small changes in Vimentin abundance in MB49 cells. The change in abundance was better evident at the 48 hour timepoint but was not significant.
